# What discontinued trials teach us about trial registration?

**DOI:** 10.1186/s13104-020-05391-w

**Published:** 2021-02-05

**Authors:** Akke Vellinga, Kathryn Lambe, Paul O’Connor, Angela O’Dea

**Affiliations:** 1 School of Medicine, National University of Ireland, Galway, Ireland; 2 Primary Care Clinical Trials Network Ireland, National University of Ireland, Galway, Ireland; 3 Irish Centre for Applied Patient Safety and Simulation (ICAPSS), School of Medicine, National University of Ireland, Galway, Ireland; 4 Royal College of Surgeons in Ireland, 121/122 St. Stephen’s Green, Dublin 2, Ireland

## Abstract

**Objective:**

Trial registries were set up to improve transparency, remove duplication, improve awareness and avoid waste. Many trials never reach the point of patient enrolment due to a myriad of reasons. The aim of this study was to investigate the reasons for and characteristics of discontinuation of trials.

**Results:**

A total of 163 discontinued trials were identified and compared to completed trials. A Survey was designed to further explore the nature and conduct of the trial. No differences in registered and categorised information was observed between discontinued and completed trials. Most trials discontinue due to patient or participant recruitment issues, often related to funding. Substantial changes to procedures or the protocol or changes to recruitment strategy were also commonly cited reasons. Survey information was available for 21 discontinued and 28 completed trials and no obvious differences could be identified. Our findings highlight the underlying problem of lack of detail, suboptimal recording, dated information and incomplete reporting of trials within a trial registry which hampers sharing and learning. To date, important progress has been made by the implementation of standards and the requirement of trials to be registered. Our review identifies areas where further improvements can be made.

## Introduction

Clinical trials are costly, time consuming, resource intensive and burdensome. Apart from the financial and resource implications, there is an ethical imperative not to expose patients to trials that are not viable or necessary. It is therefore of importance that all learnings from clinical trials are taken into account and not wasted [1]. Trial registries were set up to improve transparency, remove duplication improve awareness of trials and avoid waste in RCTs. The value and use of registries relies on complete, accurate, up to date and easily accessible information which includes the publication of protocols at study inception [2, 3]. This provides researchers with a comprehensive overview of current and previous research to avoid biased evidence, unjustified research, waste, and actual patient harm from unnecessary trials or a delay in access to beneficial treatments [4, 5].

The International Standard Randomised Controlled Trial Number (ISRCTN) register (www.ISRCTN.com) was set up in 2000. The ISRCTN register is an online searchable registry of clinical research studies which provides a unique identification number for each registered trial linked to a record of the study. Key information is recorded for each registered trial (e.g. title, primary contact, experimental hypotheses, primary and secondary outcomes, trial design, intervention). Most funding agencies and sponsors require the registration of any trial in public registries.

According to the revised Declaration of Helsinki “every clinical trial must be registered in a publicly accessible database before recruitment of first subject” [6]. Similarly, the International committee of medical Journal Editors (ICMJE) requires registration of trial methodology to protect patient interests and confidentiality and prevent unethical conduct. However, no further guidelines or requirements in relation to the results of a RCT are provided by the ICMJE [7]. While the quality of the information provided at registration was found to have improved 2004 to 2009, gaps in the provided information remained [1] including the failure to update procedures, data and results [8].

To highlight some of the issues with waste in trial research, a series of five reviews in the Lancet ‘Research: increasing value, reducing waste’, outlined how research can be made more efficient through the improved use and sharing of information [2, 9–12]. This series provided a list of 17 recommendations to help increase value, and covers issues relating to funders, regulators, journals, academic institutions and researchers. One of its suggestions to support successful replication of basic research and its application in health care, called for more ‘research on research’ [2].

An area for potential research on research which received little attention, is trials that are stopped before completion. Since the start of the ISRCTN registry, of the 14,000 registered studies, about 3% are classified as stopped i.e. discontinued. Considering that clinical trials can cost between €600,000 and €1.5 M, the waste of time and money is substantial [13].

## Main text

### Database review

Through the open access ISRCTN registry, we identified all RCT registered since 2000. On request, the ISRCTN registry provided us with a file including all online published information on all trials registered since 2000. Within this file, we identified 163 discontinued RCTs registered since 2000. To explore potential differences with completed trials we selected the next completed trial immediately registered after the discontinued as the comparison.

The information extracted from the ISRCTN registry included: the registration number, title of the trial, date of registration and overall trial status (stopped/completed). Additional information was extracted from the ISRCTN online database on age group; gender; contact person; type of study (prospective/retrospective); condition category, date registration and last edited; participant inclusion criteria; funder name; ethics approval; study design, trial setting, type and phase; start and end date; reason abandoned (if applicable); target number of participants; countries of recruitment, organisation; sponsor type. When an ISRCTN registered trial is reported as discontinued, the reason for abandonment is requested by the ISRCTN team and added to the database if obtained.

Four researchers reviewed and extracted information from 163 discontinued and 163 completed trials listed in the ISRCTN registry, immediately following the discontinued trial.

### Survey

A Survey Monkey questionnaire to obtain more information on each discontinued and completed trial was designed. The survey link was sent to the email of the contact listed on the ISRCTN online database. A mailing was send to all emails which included two reminders as well as searches to update email addresses of bounced mails.

## Results

For the database review, a total of 163 discontinued trials and 163 completed trials were evaluated. The condition category of the trials is shown in Table 1.

**Table 1:** Overview of the clinical area in discontinued and completed trials

	Discontinued		Completed	
	N	%	N	%
Cancer	27	17	17	10
Circulatory system	15	9	23	14
Digestive system	6	4	6	4
Ear, nose and throat	2	1	0	0
Eye diseases	6	4	4	3
Haematological disorders	3	2	2	1
Infections and infestations	11	7	14	9
Injury, occupational diseases, poisoning	5	3	2	1
Mental and behavioural disorders	18	11	18	11
Musculoskeletal diseases	10	6	6	4
Neonatal diseases	0	0	1	1
Nervous system diseases	6	4	11	7
Nutritional, metabolic, endocrine	8	5	20	12
Oral health	1	0	1	0
Overweight/obesity	0	0	2	1
Pregnancy and childbirth	4	3	7	4
Respiratory	3	2	5	3
Signs and symptoms	5	3	4	3
Skin and connective tissue diseases	2	1	5	3
Stroke	2	1	0	0
Surgery	16	10	2	1
Urological and genital diseases	11	7	4	3
Not applicable	2	1	9	6

There were no differences observed between discontinued and completed trial. This included the study design, which was mainly interventional (as opposed to observational), the secondary study design (e.g. cluster, cross over and parallel) and the setting (for most trials this was hospital (68%)).

The type of trial was generally testing different treatments but discontinued trials were significantly (*p* = 0.02) more often treatment trials (Table 2). Completed trials were more likely to involve diagnostic, prevention, quality of life, or ‘other types’ of trials.

**Table 2:** Overview of the type of discontinued and completed trials

	Discontinued		Completed	
	N	%	N	%
Treatment	129	79	110	67
Other trials	34	21	53	33
Diagnostic	4		5	
Prevention	12		17	
Quality of life	10		15	
Other	8		16	

The duration between the registration of the trial and discontinuation or completion showed no difference. However, the time period represents the period from registration unit the trial was stopped for discontinued trials while it represents the period from registration until completion for the completed trials.

### Evaluation of the reasons for discontinuation

The reason to discontinue a trial is reported to the ISRCTN registry as part of the process to update the database (Table 3). Most trials discontinue due to patient or participant recruitment issues (37%), often related to funding (17%) as increasing efforts to recruit patients generally has financial implications. Second to recruitment/funding issues, substantial changes to procedures or the protocol are indicated as reason for discontinuation (9%), changes to recruitment strategy or treatment were most commonly cited changes. Other issues recorded were problems with the purchase or delivery of the trial product, staff (including recruitment) and PI/internal issues, information indication equipoise, unattainable objectives, adverse events, DSMB (Data and Safety Monitoring Board) decision, regulatory issues, and superseded technology. For four trials the reason for discontinuation was not known.

**Table 3:** List of key reasons for discontinuation

Reason for discontinuation	%	N
Patient or participant recruitment issues	37	60
Related to funding	17	30
Substantial changes to procedures or the protocol	9	15
Issues related to the purchase or delivery of the trial product	8	13
Staff issues, including recruitment, and PI/internal issues	10	16
Information indication equipoise resulted in the discontinuation of seven	4	7
Objectives were no longer viable	6	9
Other reasons (adverse events (5), DSMB decision (2), Regulatory issues (2), and superseded technology (1))	6	10
Unknown	2	4

### Survey

The email survey was completed for 21 discontinued and 28 completed trials. The results of the survey provided little new information on the reason why the trial was discontinued beyond the database review due to the low number of respondents.

## Limitations

Our results indicate that the main reason for discontinuation is patient/participant recruitment, reflected in inflated target sample size estimates and unrealistic recruitment goals. Recruitment issues can delay trials and are directly related to funding. As previously identified, failure to meet recruitment targets due to overoptimistic or inaccurate estimates of recruitment [14] which could be anticipated by conducting a pilot or feasibility study [15]. Delays, due to changes in the protocol, or recruitment issues, also have an impact on researcher retention, often reported to the ISRCTN as another reason to discontinue a trial.

The existing information contained in the ISRCTN database is not detailed or specific enough to allow for the identification of reasons why a trial was discontinued. The information that was available was also difficult to interpret, similar to previous findings, as most data fields did not consist of standardised categories [8]. To allow more in-depth understanding of what factors are associated with completed trials, more information is necessary on actual recruitment, participation of a clinical trial unit or an (independent) trial steering committee. Such information can also help funders to assess if a study is delivering on its objectives.

The International Standards for Clinical Trial Registries (2012[16] and 2018[17]) designed a minimum dataset (Trial Registration Data Set) covering 20 items [18]. Our findings provide evidence of the underlying problem of lack of detail, suboptimal recording, dated information and incomplete reporting of trials which hampers sharing and learning.

Today, funding agencies as well as publishers, request the trial registration number. Information required to register a trial is rarely linked to the primary report once the research is complete resulting in the drop in the relevance of registries after the trial obtains a registration number. However, funding agencies require proof of ethical approval before funding is transferred while journal editors require checklists for the CONSORT [19]., QUOROM [20], STROBE [21] and STARD [22] statements. Consideration should be given to opportunities to bundle all this information as part of an integrated registration process necessary as part of the publication of the trial results.

An addition to these requirements could include the submission of a protocol as part of registration [23]. However, “research is not a car factory” [10] and it is important to acknowledge that adjustments to the design process are expected. Including a protocol should therefore not be a static document upload but allow to make adjustments as part of a well-documented process. Part of this process is already required by journals and included as ‘deviations from the protocol’ in the manuscript.

There are several parties responsible in ensuring access to complete and meaningful information on RCTs conducted throughout the world; the registry, the registrant and other stakeholders such as journal editors, ethical committees and funding agencies [18]. Enforcing measures designed to improve the quality of registration are suggested for journal editors and legislators, but little is known on if or how this is done [16].

Based on our study and to boost learning from others and other trials, we suggest following improvements:

Registries should consider to:

- Provide a downloadable template at the moment of registration which is required when publishing the results of the trial, similar to evidence of ethical approval or statements
- Improve the standardisation of the information provided through the use of standardised categories
- Allow registries to be fully searchable using standardised reporting.
- Extend the information gathered with up to date ethical approval information, CONSORT [19]., QUOROM [20], STROBE [21] and STARD [22] statements, actual recruitment information and other trial procedures

Funding agencies should consider to:

- Encourage and fund feasibility studies to support realistic recruitment targets

Journal editors should consider to:

- Request an up to date trial registry template as part of the manuscript submission

Our review of discontinued trials showed that not enough progress has been made to allow learning from other research. Five targeted groups (funders, regulators, journals, academic institutions and researchers) have been identified to play a part in increasing research value. We argue there is a sixth group: the trial registry should provide a common template with up-to-date information to be submitted as part of the publication of the trial results documenting each stage of the research process, from conception, through registration and publication.

## Data Availability

All data and material is available at request by mailing the corresponding author.

## Abbreviations

DSMB: Data and Safety Monitoring Board
ICMJE: International committee of medical Journal Editors
ISRCTN: International Standard Randomised Controlled Trial Number
RCT: Randomised Clinical Trial
